# Cytomegalovirus Colitis in a Patient with Severe Treatment Refractory Ulcerative Colitis

**DOI:** 10.1093/crocol/otae014

**Published:** 2024-02-28

**Authors:** Michelle M Bao, Juliana M Kennedy, Michael T Dolinger, David Dunkin, Joanne Lai, Marla C Dubinsky

**Affiliations:** Division of Pediatric Gastroenterology, Susan and Leonard Feinstein Inflammatory Bowel Disease Center, Icahn School of Medicine at Mount Sinai, NY, USA; Division of Pediatric Gastroenterology, Susan and Leonard Feinstein Inflammatory Bowel Disease Center, Icahn School of Medicine at Mount Sinai, NY, USA; Division of Pediatric Gastroenterology, Susan and Leonard Feinstein Inflammatory Bowel Disease Center, Icahn School of Medicine at Mount Sinai, NY, USA; Division of Pediatric Gastroenterology, Susan and Leonard Feinstein Inflammatory Bowel Disease Center, Icahn School of Medicine at Mount Sinai, NY, USA; Division of Pediatric Gastroenterology, Susan and Leonard Feinstein Inflammatory Bowel Disease Center, Icahn School of Medicine at Mount Sinai, NY, USA; Division of Pediatric Gastroenterology, Susan and Leonard Feinstein Inflammatory Bowel Disease Center, Icahn School of Medicine at Mount Sinai, NY, USA

**Keywords:** Cytomegalovirus colitis, ulcerative colitis, upadacitinib

## Abstract

**Background:**

Cytomegalovirus (CMV) can be reactivated in ulcerative colitis (UC), but its role in progression of inflammation is unclear. Risk factors include severe colitis and treatment with immunosuppressive medications, particularly corticosteroids and immunomodulators.

**Methods:**

We report a case of cytomegalovirus colitis in a pediatric patient with pancolitis who had been refractory to aminosalicylate, infliximab, and ustekinumab and was in clinical remission and with transmural response on upadacitinib.

**Results:**

This is a case of a 13-year-old male with UC refractory to multiple therapies who were in clinical remission on upadacitinib 30 mg daily. He developed an acute increase in symptoms and did not respond to therapy escalation with increased upadacitinib 45 mg daily for 2 weeks and prednisone for 1 week. He was diagnosed with cytomegalovirus colitis on flexible sigmoidoscopy biopsy. He was treated with intravenous ganciclovir with tapering of immunosuppressive regimen. Despite initial response, he underwent subtotal colectomy and subsequent restorative proctocolectomy with ileal pouch anal-anastomosis.

**Conclusions:**

Despite our patient having multiple risk factors for developing CMV colitis, upadacitinib may have played a role when considering its known impact on the herpes family of viruses. CMV colitis should be evaluated for in any patient who presents with worsening symptoms without evidence of other infection or response to increase in therapy.

## Graphical Abstract

**Graphical Abstract:**
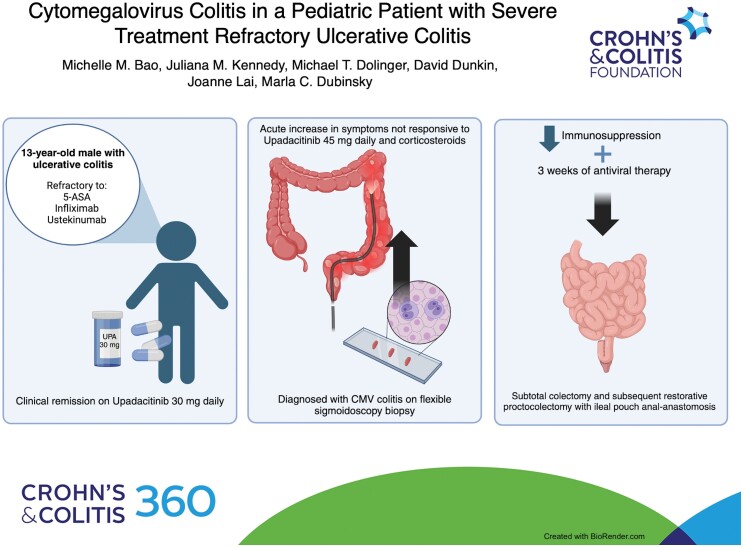

## Introduction

Cytomegalovirus (CMV) is a herpesvirus that can reactivate in immunocompromised states and lead to end-organ disease.^1^ CMV colitis has been associated with ulcerative colitis (UC) in the setting of severe colitis and immunosuppressive medications, particularly corticosteroids.^2–4^ Upadacitinib, a selective inhibitor of Janus Kinase (JAK) 1, is approved for treatment of adults 18 years and older with moderate to severe UC and Crohn disease refractory to at least 1 anti-tumor necrosis (TNF) therapy. JAK inhibitors (JAKi) are associated with increased risk of herpesvirus reactivation, particularly varicella-zoster virus (VZV). This is potentially explained by targeting interferon-mediated immune responses, which signal through JAK and inhibit VZV replication.^5^ The role of JAKi in CMV reactivation is not known.^6,7^ We present a case of CMV colitis resulting in colectomy in a 13-year-old male with severely refractive UC who developed acutely worsening symptoms while on upadacitinib 30 mg daily.

## Case Report

A 13-year-old male presented with new-onset bloody diarrhea. He tested positive for *Clostridioides difficile* toxin and received a course of vancomycin without improvement. Fecal calprotectin was 786 ug/g with pediatric UC activity index (PUCAI) score of 55. He underwent colonoscopy, which showed pancolitis. After diagnosis, he was quickly escalated to infliximab 15 mg/kg every 4 weeks in combination with weekly methotrexate. Despite brief clinical improvement with PUCAI score of 20 after induction and a therapeutic trough infliximab level in maintenance (20 μg/mL), he developed worsening frequency, rectal bleeding, and weight loss with a PUCAI score of 60. He again tested positive for *Clostridioides difficile* toxin and was started on a prolonged vancomycin taper without improvement. He transferred care to our center and was started on prednisone and ustekinumab 260 mg IV induction followed by 90 mg subcutaneous (SC) 8 weeks later. Due to a post-induction ustekinumab level of 2.9 μg/mL, his SC dosing frequency was decreased to every 4 weeks and prednisone was subsequently weaned off. After 5 months of ustekinumab treatment and trials of rectal therapies, he continued to have bloody stools. At that time, stool gastrointestinal panel and *Clostridioides difficile* toxin immunoassay were negative. He had moderate to severe inflammation of the proximal sigmoid colon to cecum on intestinal ultrasound (IUS; Table 1) with an elevated C reactive protein (CRP) of 25.4 mg/L (0–5 mg/L) and fecal calprotectin of 1464 μg/g. He was transitioned to upadacitinib 45 mg daily for 8 weeks and 30 mg daily thereafter. Twelve weeks post-initiation, he was clinically in remission with a PUCAI score of 5 with improved CRP to 11 mg/L and IUS showing transmural response, with only mild inflammation in the sigmoid and descending colon (Table 1).

**Table 1.** Monitoring of disease activity over time using intestinal ultrasound. Bowel wall thickness: normal < 3 mm; MLS, Modified Limberg score (0–III); HD, hospital day.

Intestinal ultrasound findings	5 months post ustekinumab initiation	12 weeks post upadacitinib initiation	5 months post upadacitinib initiation with acutely worsening symptoms	HD3 (after 3 days of methylprednisolone and prior to ganciclovir initiation)	HD9 (5 days post ganciclovir initiation)	Outpatient follow up (2 weeks after discharge on valganciclovir)
*Bowel wall thickness (mm)*						
Sigmoid colon	5.9	2.5	5.7	4.8	2.4	4.6
Descending colon	5.6	2.1	5.9	5.4	2.3	5.6
Transverse colon	4.2	1.3	1.1	1.1	0.8	5.7
Ascending colon/cecum	3.4	1.6	0.8	1.1	1.1	0.9
Terminal ileum	1.5	0.9	1.4	1.2	1.0	1.2
*Hyperemia by color doppler signal (MLS)*						
Sigmoid colon	III	II	III	III	II	III
Descending colon	II	I	II	III	I	III
Transverse colon	II	0	0	0	0	II
Ascending colon/cecum	II	0	0	0	0	I
Terminal ileum	0	0	0	0	0	0
Impression:	Moderate to severe inflammation from the proximal sigmoid colon to cecum	Mild inflammation in the sigmoid and descending colon with patchy increased hyperemia without increase in BWT	Severe inflammation of the sigmoid and descending colon, significantly worsened from previous	Severe inflammation of the sigmoid and descending colon, stable from previous	Significant improvement in inflammation with 50% reduction in BWT and slight improvement in hyperemia	Severe inflammation from the sigmoid to mid-transverse colon, worsened from previous

Five months after starting upadacitinib and 9 months off corticosteroids, his symptoms acutely worsened with 16 bloody bowel movements daily and weight loss due to reduced appetite. Stool infectious studies were negative. Symptoms persisted despite upadacitinib escalation from 30 to 45 mg daily for 1 week. IUS revealed severe inflammation in the sigmoid and descending colon only (Table 1). Laboratory studies revealed marked rise in CRP to 151.9 mg/L, borderline elevated white blood cell count of 10.5 × 10^3^/uL with lymphocytosis to 3.1 × 10^3^/uL and normal neutrophil count to 5.7 × 10^3^/uL, hypoalbuminemia to 2.9 g/dL, rising fecal calprotectin, and normal liver enzymes (Table 2). He received a previously planned 300 mg induction dose of IV vedolizumab, IV methylprednisolone 40 mg, and IV fluid hydration. Oral prednisone 40 mg daily was initiated, and upadacitinib 45 mg was continued.

**Table 2.** Laboratory values from time of symptom worsening, during first hospital admission, outpatient follow-up, and day of subtotal colectomy. HD, hospital day; CRP, C reactive protein; ESR, erythrocyte sedimentation rate; ALT, alanine aminotransferase; AST, aspartate aminotransferase; CMV, cytomegalovirus; PCR, polymerase chain reaction.

Laboratory values (reference range)	Day of outpatient IV vedolizimab and methylprednisolone	HD1 (7 days after outpatient infusion)	HD3 (day of flexible sigmoidoscopy)	HD4 (intravenous ganciclovir initiation)	HD6	HD9	HD11	HD12 (discharge on oral valganciclovir)	Outpatient follow-up (2 weeks after discharge)	Day of subtotal colectomy
White blood cell (4.4–10.5 × 10E3/uL)	10.5	12	14	11.3	13.5	12.5	10.1	9.4	10.5	12.4
Neutrophil (2–7.1 × 10E3/uL)	5.7	9.9	10.4	8.1	8.0	7.1	5.6	7.3	7.5	7.9
Lymphocyte (1–2.8 × 10E3/uL)	3.1	1.3	2.2	2.4	4.2	4.2	3.6	1.6	2.1	2.9
Monocyte (0.4–1.3 × 10E3/uL)	1.4	0.7	1.2	0.6	0.9	0.9	0.7	0.4	0.8	1.5
Eosinophil (0–0.6 × 10E3/uL)	0.2	0.0	0.0	0.1	0.2	0.2	0.1	0.1	0.0	0.0
Basophil (0–0.2 × 10E3/uL)	0.0	0.0	0.1	0.0	0.0	0.0	0.0	0.0	0.0	0.0
Hemoglobin (10.7–16 g/dL)	12.4	12.2	11.8	12.1	10.9	12.7	11.5	12.3	11.2	9.9
Platelet (150–450 × 10E3/UL)	609	425	512	542	436	449	361	416	714	564
ESR (0–30 mm/h)	60	96			58	56		50	100	
CRP (0–5 mg/L)	151.9	151.6			41	25.4	32.3	42.3	84.9	
Albumin (3.5–4.9 g/dL)	2.9	2.5	2.4		2.1	2.3	2.2	2.4	2.6	
ALT (1–45 U/L)	7	51	82			71	70	76	11	
AST (1–35 U/L)	9	24	22			15	24	21	10	
CMV quantitative PCR (not detected IU/mL)				3710		1800		1100	<34.5	
Fecal calprotectin (ug/g)		3367				3365	914			

**Figure 1.**
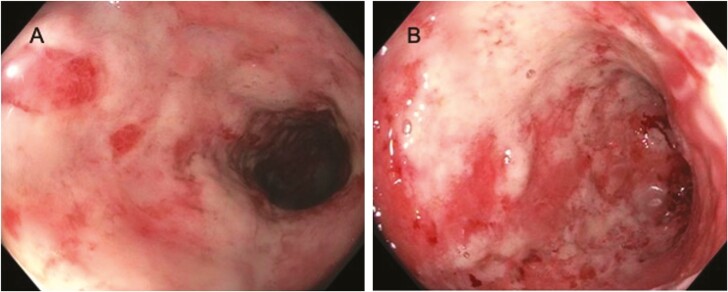
Flexible sigmoidoscopy images demonstrating severe Mayo 3 inflammation with deep ulcerations, denuded mucosa, and spontaneous bleeding from the rectum to the splenic flexure. (A) Descending colon. (B) Splenic flexure.

Due to lack of any symptomatic improvement on 1 week of corticosteroids, he was admitted for hydration, further evaluation and consultation with colorectal surgery. Laboratory studies showed persistently elevated CRP to 151.6 mg/L, rise in white blood cell count to 12 × 10^3^/uL with neutrophilia to 9.9 × 10^3^/uL and normal lymphocyte count to 1.3 × 10^3^/uL, worsening hypoalbuminemia to 2.5 g/dL, and alanine aminotransferase (ALT) to 51 U/L (Table 2). IV methylprednisolone 16 mg twice daily was started and upadacitinib 45 mg daily was continued. After no clinical improvement and continued severe inflammation in the sigmoid and descending colon on inpatient IUS (Table 1), flexible sigmoidoscopy on hospital day (HD) 3 revealed Mayo 3 inflammation with denuded mucosa from the rectum to splenic flexure (Figure 1). On HD4, histopathology resulted with rare viral cytopathic effect and presence of positive immunohistochemical (IHC) stained cells (up to 2 per high-power field and up to 5 per tissue fragment) in all 6 biopsies obtained, and serum CMV PCR was elevated at 3710 IU/mL (reference range: Not detected IU/mL). IV ganciclovir 5 mg/kg twice daily was started. In terms of immunosuppression, upadacitinib was discontinued and IV methylprednisolone was decreased by 50% to 16 mg daily. On HD8 (4 days of ganciclovir), he was having 3–5 partially formed stools per day with less blood and was transitioned to oral prednisone 20 mg daily. On HD9, CMV PCR decreased to 1800 IU/mL and CRP decreased (Table 2). IUS showed significant improvement with 50% reduction in BWT and improved hyperemia in the sigmoid and descending colon (Table 1). After 7 days of IV ganciclovir, he has discharged on oral valganciclovir 900 mg daily to complete 3 weeks of antiviral therapy and oral prednisone 20 mg daily.

Near the end of his course of oral valganciclovir as an outpatient, he developed increased bloody stools. After 2.5 weeks of antiviral therapy, he was restarted on upadacitinib 45 mg daily and continued on oral prednisone 20 mg daily without improvement. IUS performed 2 weeks later revealed worsening inflammation and disease extension to the mid-transverse colon (Table 1). Laboratory studies revealed rise in CRP, persistent hypoalbuminemia to 2.6 g/dL, while ALT had normalized, and CMV DNA PCR was < 34.5 IU/mL (Table 2). He was readmitted and underwent subtotal colectomy with end ileostomy on HD2. Histopathology of the surgical specimen showed severely active chronic inflammation with no viral cytopathic effect and a single positively stained IHC cell. At his postoperative follow-up appointment 4 weeks later, he was in clinical remission with a 15 lb. weight gain and was weaned off of prednisone. Restorative proctocolectomy with ileal pouch anal-anastomosis was performed 12 weeks after subtotal colectomy and he has continued to do well post ileal pouch anal-anastomosis.

## Discussion

We report a case of CMV colitis in a patient with severe UC refractory to multiple therapies after a brief period of clinical remission on upadacitinib. The role of CMV in UC severity and disease progression is unclear. Risk factors for CMV infection in IBD include older age (>30 years), pancolitis, histologic inflammation, and immunosuppressive therapy.^2–4,8^ Studies have attempted to address issues raised when treating a patient with UC and suspected CMV colitis, including optimal methods of diagnosis, treatment with antiviral therapy, and impact on UC disease outcomes, such as response to immunosuppressive therapies and need for colectomy.

Immunosuppressive therapies have been variably linked to CMV reactivation. Corticosteroids, chronic low-dose and short-term high doses, and thiopurines have both been shown to increase risk.^9^ Anti-TNF agents do not appear to increase the risk of CMV reactivation, and 2 studies showed that giving anti-TNF concurrently with antiviral therapy to patients with severe UC and CMV infection does not appear to affect rates of colectomy compared to antiviral therapy alone.^9,10^ There is limited data on the effects of other biological and small molecule therapies in CMV reactivation. In the UNIFI trial with ustekinumab, there were 2 reported cases of CMV during maintenance therapy, but these patients were receiving concomitant corticosteroids.^11^ Safety data from vedolizumab suggests no increased risk, although a case has been reported of a patient who developed CMV colitis on vedolizumab monotherapy and responded to antiviral therapy.^12^

JAKi are known to increase the risk of herpes zoster from VZV reactivation, but their effect on CMV reactivation in UC is not clear.^6,7^ A single-center retrospective study of tofacitinib use in 58 IBD patients reported 2 cases of CMV colitis, although patients were on concomitant corticosteroids.^13^ One case of CMV colitis was reported in the phase 3 upadacitinib U-ACCOMPLISH induction study on 45 mg daily dosing, which was nonserious and did not lead to treatment discontinuation.^14^ CMV reactivation has been reported in other autoimmune diseases, including CMV retinitis in patients with rheumatoid arthritis being treated with tofacitinib and upadacitinib.^15,16^

While there is no standard definition for CMV colitis, the presence of inclusion bodies on hematoxylin and eosin (H&E) stain, positive IHC stained cells in colonic tissue, and/or tissue PCR from endoscopic biopsy is recommended for diagnosis.^12,17^ CMV tissue load by PCR thresholds or histologic criteria appears to be predictive of whether CMV is more likely a pathogenic driver or an incidental finding in the setting of severe colitis.^17^ Patients who meet study-specific designated criteria for high-grade CMV colitis have been reported to have higher rates of colectomy and are less likely to respond to corticosteroid treatment.^2–4,18,19^ Colonic tissue viral load has also been suggested to predict response to antiviral therapy. In studies that analyzed outcomes by subgroup, patients with high-grade CMV colitis (≥5 inclusions in any single fragment in Jones et al, presence of viral inclusion bodies on H&E and special IHC stains in Nguyen et al, CMV DNA load >250 copies/mg in Roblin et al) were more likely to respond to antiviral treatment and have decreased rates of colectomy.^2,18,20^

Our patient, who was in clinical remission with transmural response on IUS, developed acute worsening symptoms on upadacitinib monotherapy after a 9-month corticosteroid hiatus and was diagnosed with high-grade CMV colitis based on histopathology. It is possible that upadacitinib led to CMV reactivation, but in this patient who had a particularly refractory course, it is also possible it occurred due to worsening colitis from loss of response to JAKi or initiation of corticosteroids. While he ultimately required subtotal colectomy, his CMV infection was treated based on lack of viral cytopathic effect from his surgical specimen and minimally detectable blood CMV PCR after antiviral therapy. While patients with high-grade CMV colitis seem to benefit from antiviral therapy, colectomy rates remained high, ranging from 33% to 44%.^2,18,20^ Optimal management of immunosuppression in CMV colitis in UC remains uncertain. The European Crohn’s and Colitis Organization guidelines recommend tapering of corticosteroids,^21^ but there are currently no recommendations for JAKi. In our patient, upadacitinib was discontinued while initiating antiviral therapy and tapering corticosteroids. CMV colitis should be considered in any UC patient who develops acute worsening symptoms without evidence of other infection and is nonresponsive to increase in anti-inflammatory therapy.

Informed consent was obtained from the parents of the patient to publish the information and images in this case report.

No new data was created or analyzed for this case report.
